# Supplementary material for: Polyacetylene Isomers Isolated from Bidens pilosa L. Suppress the Metastasis of Gastric Cancer Cells by Inhibiting Wnt/β-Catenin and Hippo/YAP Signaling Pathways
Source: Molecules. 2023 Feb 15;28(4):1837. doi: 10.3390/molecules28041837 (PMC9962988; doi:10.3390/molecules28041837)
Supplement: Supplementary file 1 [file molecules-28-01837-s001.zip › molecules-2165904-supplementary.pdf]

Supplementary materials

1. NMR spectra (Compound No: BPP-36-2 for Polyacetylene 1, BPP-31-1 for Polyacetylene 2).

Polyacetylene 1 ^{13}C-NMR spectrum

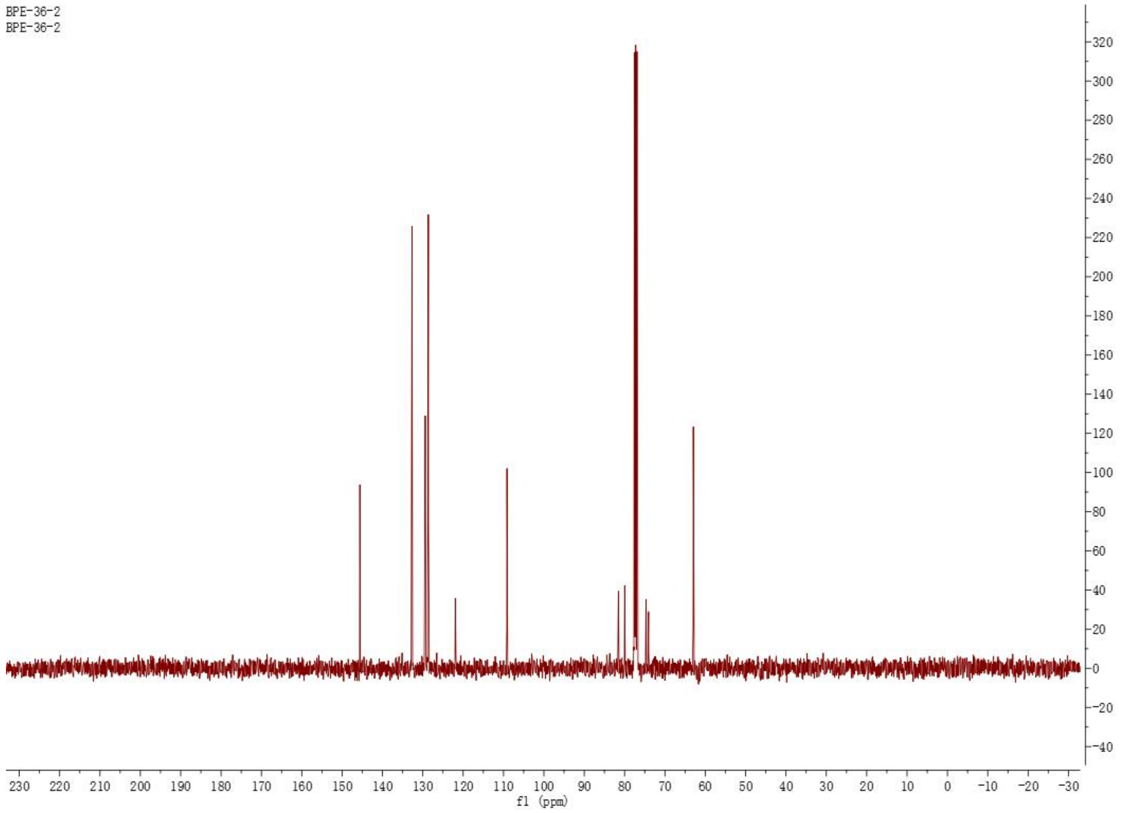

Polyacetylene 1 ^{1}H-NMR spectrum

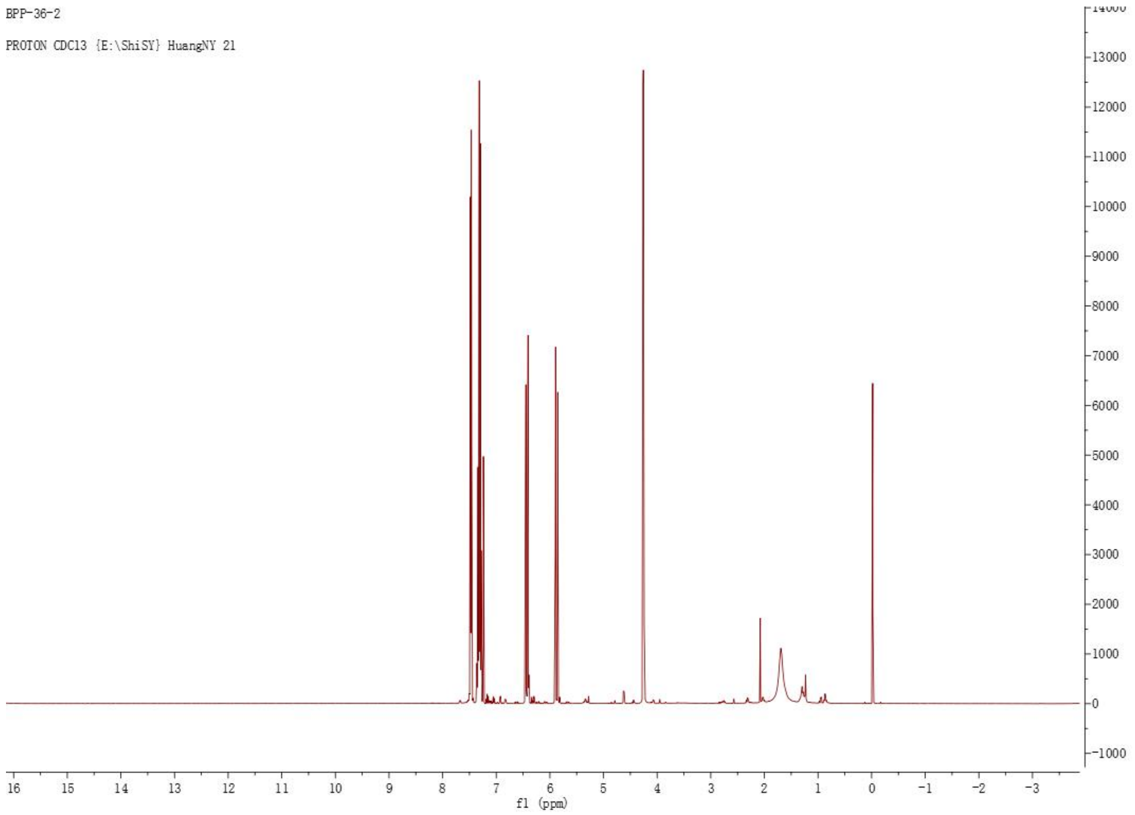

Polyacetylene 2 ^{13}C-NMR spectrum

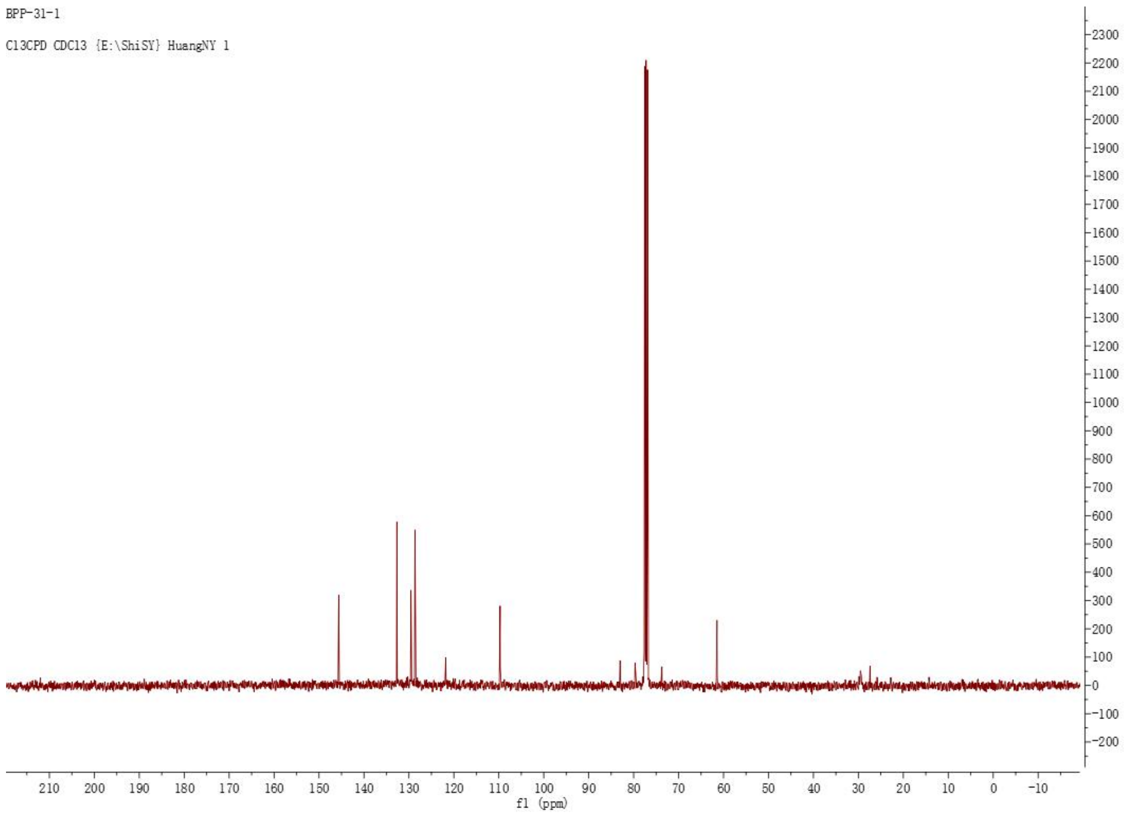

Polyacetylene 2 ^{1}H-NMR spectrum

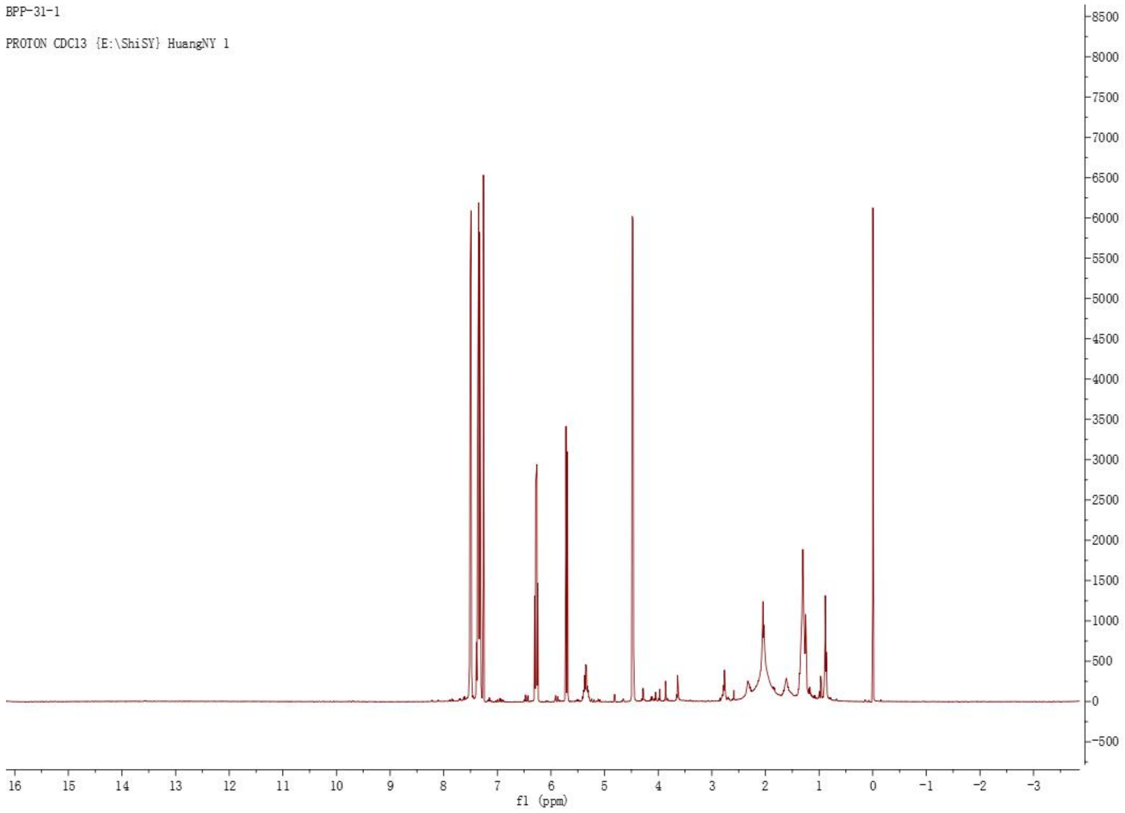

Polyacetylene 2 DEPT spectrum

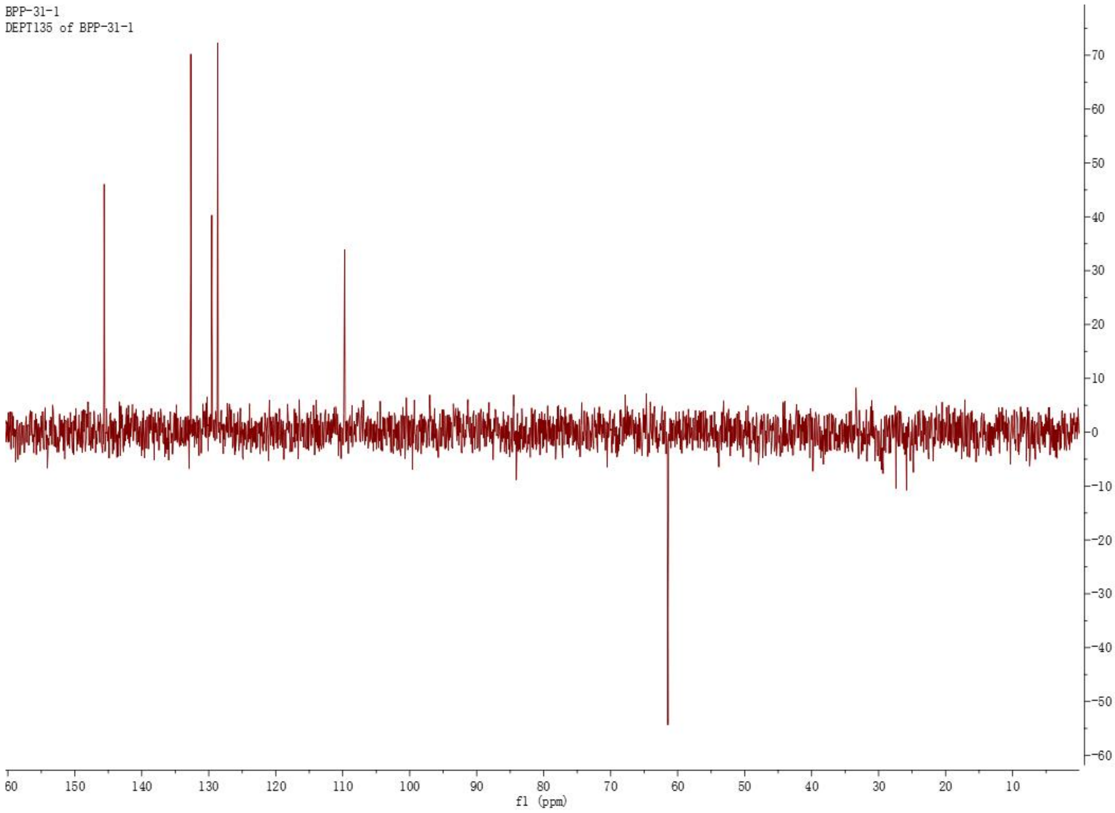

2. The culture medium used for each cell lines.

Human gastric carcinoma HGC-27 cells, human gastric carcinoma BGC-823 cells, human gastric carcinoma NCI-N87 cells, human cervical cancer Ca Ski cells, and human nasopharyngeal carcinoma CNE-2 cells were maintained in RPMI-1640 medium.

Human breast cancer MDA-MB-231 cells, human hepatocellular carcinoma HepG2 cells, human colon cancer HCT-116 cells,human pancreatic cancer PANC-1 cell, human lung cancer A549 cells, human gastric epithelial GES-1 cells, and Madin-Darby canine kidney MDCK cells were maintained in DMEM medium.
